# miR-181b-5p May Regulate Muscle Growth in Tilapia by Targeting Myostatin b

**DOI:** 10.3389/fendo.2019.00812

**Published:** 2019-12-03

**Authors:** Zaoya Zhao, Xiaozheng Yu, Jirong Jia, Guokun Yang, Caiyun Sun, Wensheng Li

**Affiliations:** State Key Laboratory of Biocontrol, Institute of Aquatic Economic Animals and Guangdong Province Key Laboratory for Aquatic Economic Animals, Guangdong Provincial Engineering Technology Research Center of Healthy Breeding in Important Economic Fish, School of Life Sciences, Sun Yat-sen University, Guangzhou, China

**Keywords:** myostatin, tilapia, microRNA, growth, muscle, primary muscle cells

## Abstract

**Background:** Myostatin (Mstn), a member of the TGF-β superfamily, is a negative regulator of skeletal muscle mass in mammals. Precise regulation of Mstn expression is important for somite growth in fish. MicroRNA (miRNA), a type of small non-coding RNA, regulates gene expression at the post-transcriptional level and participates in various physiological functions. A growing amount of evidence has emphasized the importance of miRNA in the development of skeletal muscle.

**Aims:** This study aims to study how miRNAs regulate *myostatin b* (*mstnb*) post-transcriptionally in tilapia.

**Methods/Results:** *Mstnb* 3′ UTR sequences were obtained, and the results of tissue distribution showed that *mstnb* was expressed in several tissues, including brain, white muscle, gut, and adipose tissue. A total of 1,992 miRNAs were predicted to target *mstnb* in tilapia using bioinformatics, and a dual-luciferase reporter experiment confirmed that miR-181a/b-5p, miR-30-3p, miR-200a, and miR-27a were the target miRNAs of *mstnb*. Mutagenesis of the miR-181b-5p binding sites of *mstnb* significantly increased the luciferase signal compared to the wild-type *mstnb*. In tilapia primary muscle cells, overexpression of miR-181b-5p led to the downregulation of MSTNb expression, and the inhibitory effect of MSTNb on the downstream genes was dismissed, while inhibition of miR-181b-5p could reverse these phenomena.

**Conclusion:** Taken together, our results suggested that miR-181b-5p could promote the growth of skeletal muscle by decreasing the MSTNb protein level in tilapia.

## Introduction

Genetic Improvement of Farmed Tilapia (GIFT), a freshwater fish with a fast growth rate and high disease resistance, is a popular aquaculture fish worldwide that provides premium protein for people. Since the major edible part of the fish is skeletal muscle, the fish growth performance is mainly determined by the development of skeletal muscle. Skeletal muscle development is an accurate process that is regulated by positive factors, including muscle-specific myogenic regulatory transcription factors (MRFs) (1) and negative factors such as myostatin (Mstn).

Myostatin, a member of the transforming growth factor β (TGFβ) superfamily, is regarded as a specific muscle negative regulatory factor (2). Mstn, a secretory protein in skeletal muscle, is composed of 376 amino acids, including a signal peptide, an N-terminal precursor peptide, and a C-terminal mature peptide that contains nine conserved cysteine amino acids (3). In mammals, *mstn* is specifically expressed in skeletal muscle, while *mstn* is widely distributed in teleosts. In addition, *mstn* has several different types in teleosts as a result of gene duplication. According to the genome listed in the NCBI, for example, *mstn*-1 (or *mstnb*) and *mstn*-2 (or *mstna*) exist in Nile tilapia. Tissue distribution revealed that *mstnb* is mainly expressed in the brain, eye, gill, gut, and skeletal muscle in Nile tilapia (4). *Mstn* can inhibit the growth of skeletal muscle in mammals, but its functions in teleosts are not clear. In tilapia, scientists reported that prolonged fasting reduced the mRNA level of *mstn*, but short-term fasting elevated the mRNA level (5, 6). Moreover, the proliferation was inhibited and differentiation was consequently activated after MSTN-1 incubation of the myosatellite cells in rainbow trout (7). Regardless, Mstn is an important factor for skeletal muscle in teleosts.

Due to the strong inhibitory effect on muscle growth in mammals, it is particularly important to regulate the expression of Mstn. On the one hand, Mstn can be tightly regulated at the transcriptional level. E-Box sequence motifs, the canonical binding site for the basic Helix-Loop-Helix (bHLH) transcription factors (MyoD, Myogenic Differentiation Antigen; Myf5, Myogenic factor 5; and MyoG, Myogenin), were found in the *mstn* promoter (8, 9). The putative myocyte enhancer factor 2 (mef2) transcription factors binding motifs were also observed in the *mstn* promoter (9–12), and they were shown to increase Mstn expression in myoblasts (11). On the other hand, Mstn can be regulated at the post-transcriptional level. MiRNAs, a type of short non-coding RNA, inhibit translation or degrade the mRNA by binding to the 3′ UTR of targeted mRNAs (13). MiRNAs take part in numerous developmental processes, including the development of skeletal muscle (14, 15). Several muscle-specific miRNAs, including miR-1, miR-133a, miR-133b, and miR-206, were identified and shown to regulate myogenesis in mammals (16). For example, miR-1 and miR-206 affected muscularity by targeting *mstn* in Texel Sheep due to a mutation in the 3′ UTR (17). There is a complex regulatory network between miRNAs and genes; one gene can be regulated by several miRNAs and one miRNA can regulate multiple genes (18). In mammals, miR-27 was reported to regulate *mstn* expression by directly targeting the 3′ UTR (19–22). For example, MSTN could inhibit its own expression by upregulating miR-27 expression through a smad3-dependent mechanism (21). In teleosts, only miR-181a-5p was reported to target the *mstn* 3′ UTR in *Siniperca chuatsi* (23). MiRNAs regulating the expression of Mstn post-transcription levels have attracted more attention in recent years. However, it is unclear whether miRNA regulates Mstn in tilapia.

In our previous study, a deep sequencing of the Nile tilapia miRNA transcriptome was conducted in our lab (24). In this study, the candidate miRNAs that target *mstn* were predicted based on the miRNA transcriptome database. We screened the miRNAs that targeted *mstn* using the dual-luciferase reporter system and verified the regulation of miRNA on *mstn* in tilapia primary muscle cells. The objective of this study was to find miRNAs that target *mstn* and regulate the growth of tilapia. Clarifying the regulatory mechanism of *mstn* using miRNA for skeletal muscle growth would help deepen the understanding of tilapia growth. In addition, it is a new paradigm to study miRNA in fish with economic value. This could increase economic benefits and make an important contribution to the aquaculture industry.

## Materials and Methods

### Experimental Fish and Tissue Sample Preparation

Tilapia were obtained from the local farm of Guangdong Tilapia Breeding Farm. They were maintained in a water circulation system with water temperature at 28°C under a 12/12 h light/dark photoperiod. The fish were fed to satiety daily with commercial extruded feed (Tongwei, Foshan, China). The time of domestication was longer than 1 week. They were narcotized with eugenol before decollating. Skeletal muscle samples were collected from fish weighting 6–8 g.

### Prediction of *mstnb*-Binding miRNAs

First, the sequences of the *mstnb* and *mstna* 3′ UTR were obtained using PCR with KOD neo plus (TOYOBO, Osaka, Japan). To predict miRNAs that potentially bind to *mstnb*, a tilapia miRNA transcriptome was conducted (data not shown) and the PITA targets (http://genie.weizmann.ac.il/pubs/mir07/mir07_prediction.html) were queried (24).

### Luciferase Assay

A recombined psiCHECK2 vector (Promega, Madison, USA) containing the *mstnb* 3′ UTR downstream of the stop codon of the Ranilla luciferase gene was constructed, and the firefly luciferase was used as a reference gene. The mutant *mstnb* 3′-UTR reporters were created using site-directed mutagenesis at the binding sites of the predicted miR-181b-5p, and primers were designed using Primer X (http://www.bioinformatics.org/primerx/). These reporters and miRNA mimics (synthesized by GenePharma, Shanghai, China) were co-transfected into HEK 293T cells, and the relative luciferase activity was detected using the Luciferase Assay Systems kit (Promega, USA) according to the manufacturer's protocol. All primers are listed in Table S1.

### Tissue Distribution of *mstnb/a* mRNA and miR-181b-5p in Tilapia

For miR-181b-5p cloning, the specific primers were designed according to the miRNA transcriptome, and the sequence of miR-181b-5p was further verified. For tissue distribution, total RNA was extracted from the tissue samples of the telencephalon, diencephalon, cerebellum, medulla oblongata, spinal cord, hypothalamus, pituitary, gill, heart, liver, spleen, stomach, foregut, midgut, hindgut, adipose tissue, red muscle, white muscle, testis, and kidney of three adult male tilapia (BW 150–180 g). All samples were snap-frozen in liquid nitrogen once removed, followed by storage at −80°C until RNA extraction. After RNA extraction and reverse transcription, the tissue distribution of *mstnb/a* and miR-181b-5p was assayed using real-time PCR.

### Real-Time PCR

Total RNA from each well (*n* = 3–4) was extracted from primary muscle cells using Trizol reagent (Invitrogen, Carlsbad, CA, USA), and the RNA concentration was determined using a Nanodrop200C spectrophotometer (Thermo Scientific, Waltham, USA). A total of 1 μg of total RNA was reverse-transcribed into cDNA with the M-MLV Reverse Transcriptase (Life Technology, Carlsbad, USA), and the PCR reaction was amplified with the Thunderbird SYBR Green qPCR Mix (TOYOBO, Japan) according to the manufacturer's protocol. For mRNA quantitative analysis, β-actin was detected as the internal normalization control. Specifically, miRNA stem-loop primers were used in reverse transcription and U6 snoRNA was used as the internal control. Gene expression was normalized against the expression of the control using the comparative Ct method (25). Each experiment was repeated three times independently.

### Preparation of the Polyclonal Antibody Against Recombinant MSTNb

First, the sequence of the tilapia *mstnb* ORF without the signal peptide was subcloned into the pET-32a vector. The recombinant expression vector pET-32a-rMSTNb was transformed into *Escherichia coli* BL21. The method of recombinant protein expression was based on previous reports (26). Briefly, the cells were induced using 1 mM IPTG for 4 h at 37°C when the optical density (OD 600) reached 0.5–0.6. RMSTNb (recombinant MSTNb) was purified by cutting the object tape in SDS-PAGE. Western blots targeting a 6 × His-tag were used to assess the production of the purified protein. Second, the purified rMSTNb was injected into a New Zealand White rabbit for 4 weeks to produce the polyclonal antibody. The serum was sampled and stored at −80°C after the final immunization. All of the procedures involving the polyclonal antibody and specificity determination were based on previous reports (26).

### Immunoblotting

To detect the MSTN protein expression, rMSTNb protein was expressed using the prokaryotic expression system and a rabbit polyclonal antibody against rMSTNb was developed. Before protein extraction, PMSF (phenylmethanesulfonyl fluoride) and protease inhibitor (Beyotime, Nantong, China) were added to a cell lysis reagent radio immunoprecipitation assay buffer (Beyotime, China) at a ratio of 1:100. First, the concentration of protein was determined, and 10 μg total protein was separated on an SDS-PAGE and transferred to a polyvinylidene fluoride membrane (0.45 μm, Millipore, New York, USA). Next, 5% BSA (dissolved in TBST) was used to block the membrane for an hour at room temperature, and the specific antibody was incubated at 4°C overnight. The internal control was β-actin (Proteintech, Chicago, USA) or GAPDH (Cell Signaling Technology, Boston, USA). Third, HRP-conjugated goat anti-rabbit or anti-mouse IgG antibody (Boster, Wuhan, China) was incubated for an hour at room temperature after washing the membrane three times (10 min each). Finally, the membranes were washed, and the immunoreactivity was determined by an enhanced chemiluminescence ECL detection kit (Amersham, Buckinghamshire, UK). The gray intensity analysis was conducted using Image J 1.45 (NIH, Bethesda, USA).

### Primary Muscle Cell Isolation and Identification

The primary muscle cell culture was conducted as previously described with some modifications (27). Briefly, white muscle was obtained from the latero-dorsal muscle of juvenile tilapia (6–8 g, *n* = 25–30) and collected in an ice-cold isolation medium (DMEM, 9 mM NaHCO_3_, 20 mM HEPES, 100 U/ml penicillin, 100 U/ml streptomycin, and 15% horse serum). After removing the remaining red muscle and skin, the samples were sliced and hydrolyzed with collagenase (2 mg/ml, Sigma-Aldrich, St. Louis, MO, USA) at 28°C for 20 min. After washing twice with washing medium (isolation medium without horse serum), trypsin (1 mg/ml, Sigma-Aldrich, USA) was used to digest the remaining sample at 28°C for 20 min. This mixture was diluted with additional isolation medium (1:4) to neutralize the digestion of trypsin. After centrifugation, the cells were resuspended with complete medium (washing medium with 10% FBS) and filtered through a sterile nylon sieve (100-, 200-, and 400-mesh). Then, the myosatellite cells were collected in complete medium, and seeded onto 24-well or 12-well plates (Corning, NY, USA) at a density of 4 × 10^5^ cells/cm^2^ for different experiments. After 24 h, the adhered myosatellite cells were covered with fresh complete medium. To further verify the adhered myosatellite cells, an immunofluorescence experiment with MyoD (ab203383, Abcam, Cambridge, UK) and MyoG (M-225; Santa Cruz Biotechnology, Santa Cruz, USA) antibody was conducted as previously described (27). Both MyoD and MyoG are myogenic regulatory factors with dynamic expression in the process of muscle differentiation (28).

### Regulation of miR-181b-5p *in vitro*

To detect the transfection efficiency, we transfected miR-181b-5p (with or without CY3-label, synthesized by GenePharma, China) into primary muscle cells using Lipofectamine3000 (Life Technologies, USA) at 80 nM for 24 h, and the inverted fluorescent microscope ECLIPSE Ti-E (Nikon, Tokyo, Japan) was used to observe the fluorescence. Transfection efficiency was defined as the ratio of cells in red (CY3-labeled) and number of cells in blue (DAPI stained). MiRNA mimics and antagomir (synthesized by GenePharma, China) were used in the overexpression and inhibition experiments. The negative control was a scrambled RNA duplex that was not homologous to the tilapia genome. All oligonucleotides were 2′-OMe modified, and the end of the antagomir was conjugated to cholesterol. MiRNA mimics were transfected into the dispersed cells at 80 nM for 24 h, while antagomir was incubated at 100 nM for 24 h, and cells were harvested for RNA extraction or western blot. Each experiment was repeated three times independently.

### Statistical Analysis

Data are expressed as the means ± SEM unless otherwise stated. Statistical significance was assessed by one-way ANOVA followed by Bonferroni's multiple comparison tests. Statistical significance was defined as *p* < 0.05. *p* < 0.05 was noted with ^*^p < 0.01 with ^**^, and p < 0.001 with ^***^.

## Results

### Molecular Cloning and Tissue Distribution of *Mstnb* and *Msnta* in Tilapia

**Figure 1:**
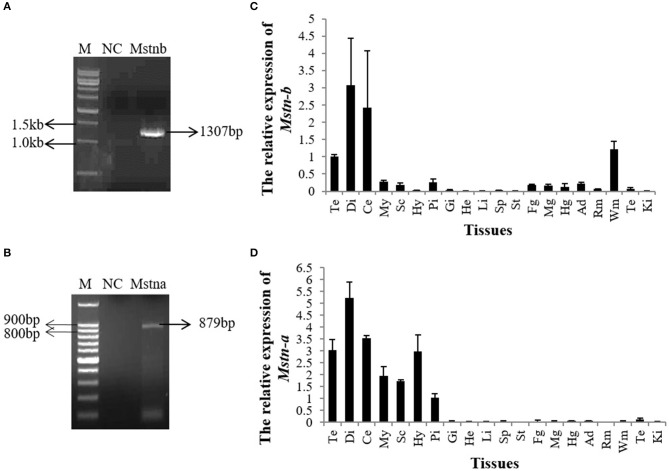
The expression pattern of tilapia *mstnb* and *mstna* by qPCR. *Mstnb* and *mstna* clones were identified by PCR in **(A)** tilapia brain and **(B)** white muscle. Relative mRNA expression of **(C)** *mstnb* and **(D)** *mstna* in the tissues of tilapia (*n* = 3, males). Te, telencephalon; Di, diencephalon; Ce, cerebellum; My, Myelencephalon; Sc, spinal cord; Hy, Hypothalamus; Pi, pituitary; Gi, gill; He, Heart; Li, Liver; Sp, spleen; St, stomach; Fg, foregut; Mg, midgut; Hg, hindgut; Ad, adipose tissue; Rm, red muscle; Wm, white muscle; Te, testis; Ki, Kidney. Bar: mean ± SEM.

The 3′ UTRs of *Mstna* and *Mstnb* were cloned. The results showed that the *Mstnb* 3′ UTR was 1,307 bp, which was consistent with the *Mstnb* sequences in the Nile tilapia genome (Figure 1A and Data Sheet S1). The *Mstna* 3′ UTR was 879 bp, which differed from *Mstna* sequences in the Nile tilapia genome (Figure 1B and Data Sheet S1). QPCR analysis showed that *Mstnb* mRNA was abundantly expressed in the telencephalon, diencephalon, cerebellum, and white muscle, and weakly expressed in the pituitary and gut (Figure 1C). However, *Mstna* mRNA was only expressed in the brain of tilapia (Figure 1D). Therefore, our present study was focused on *Mstnb*.

### Screening of miRNAs Targeting *Mstnb*

**Figure 2:**
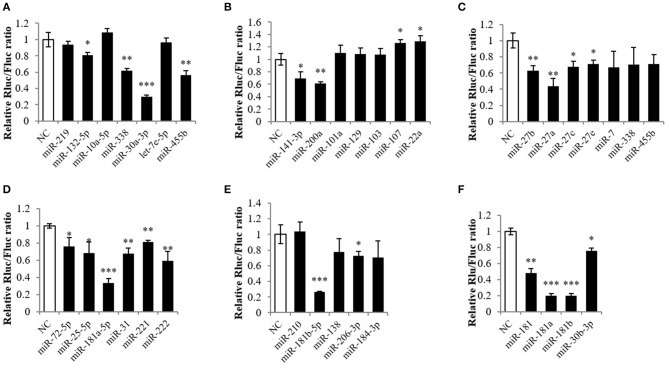
Screening the targeting miRNA of *Mstnb*. Dual luciferase assay with psiCHECK2-*Mstnb* and predicted target miRNA, and the y-axis is represented by the ratio of ranilla luciferase to firefly luciferase. **(A–F)** Showed different miRNA may target mstnb. **(A)** miR-338, miR-30a-3p, and miR-455b; **(B)** miR-141-3p and miR-200a; **(C)** miR-27b, miR-27a, and miR-27c; **(D)** miR-181a-5p, miR-31, miR-221, and miR- 222; **(E)** miR-181b-5p and miR-206-3p; **(F)** miR-181, miR-181a/b, and miR-30b-3p. Bar: mean ± SEM. ^*^P < 0.05, ^**^P < 0.01, and ^***^P < 0.001 One-way ANOVA analysis, *n* = 3.

To predict miRNAs that potentially target *Mstnb*, the PITA prediction program was employed based on the database of the tilapia miRNA transcriptome. After the prediction, 1,992 miRNAs were predicted to target the 3′ UTR of *Mstnb* (data not shown). Subsequently, a preliminary screening was carried out with the principle of conservation, and, finally, 32 miRNAs with high scores were selected to conduct different experiments. The *Mstnb* 3′ UTR was subcloned into the psiCHECK2 reporter plasmid and used in a dual luciferase reporter experiment. The results showed that miR-30a-3p, miR-181a-5p, and miR-181b-5p downregulated the relative Renilla/Firefly luciferase ratio (Rluc/Fluc) of the *Mstnb* 3′ UTR (*P* < 0.001) (Figures 2A,D,E); miR-338, miR-455b, miR-200a, miR-27b, miR-27a, miR-31, miR-221, and miR-222 downregulated the Rluc/Fluc (*P* < 0.01) (Figures 2A–D); and miR-132-5p, miR-141-3p, miR-107, miR-22a, miR-27c, miR-27e, miR-72-5p, miR-25-5p, and miR-206-3p downregulated the Rluc/Fluc (*P* < 0.05) (Figures 2A–E). Specially, overexpression of miR-181a/b-5p and miR-30a-3p decreased the Rluc/Fluc by more than 50%. The other members of miR-181 and miR-30 were also included in the dual luciferase reporter experiment. The results showed that miR-181, miR-181a, miR-181b, and miR-30b-3p also downregulated the Rluc/Fluc (Figure 2F). Among these miRNAs, miR-181 was reported to be abundantly expressed in muscle and related to skeletal muscle growth in mammals (29). Thus, miR-181 was chosen for further study.

### miR-181b-5p Directly Targets the *Mstnb* 3′ UTR

**Figure 3:**
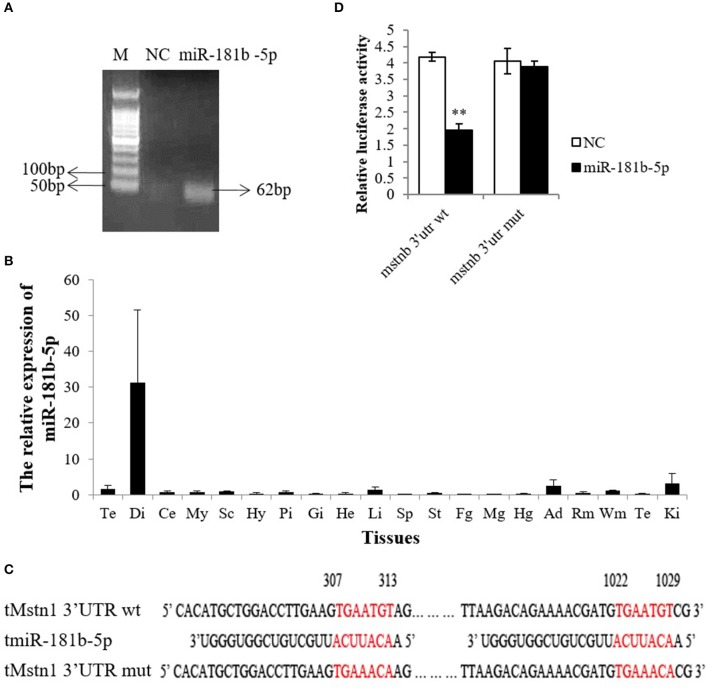
miR-181b-5p directly targeted *Mstnb* 3′UTR. **(A)** MiR-181b-5p clones were identified by PCR in tilapia brain. **(B)** Relative mRNA expression of miR-181b-5p in the tissues of tilapia (*n* = 3, males). Te, telencephalon; Di, diencephalon; Ce, cerebellum; My, Myelencephalon; Sc, spinal cord; Hy, Hypothalamus; Pi, pituitary; Gi, gill; He, Heart; Li, Liver; Sp, spleen; St, stomach; Fg, foregut; Mg, midgut; Hg, hindgut; Ad, adipose tissue; Rm, red muscle; Wm, white muscle; Te, testis; Ki, Kidney. **(C)** Complete complementarity between *Mstnb* 3′ UTR and miRNA (*Mstnb* 3′ UTR wt) and incomplete complementarity (*Mstnb* 3′ UTR mut). **(D)** Dual luciferase assay with psiCHECK2-*Mstnb* 3′ UTR wt or psiCHECK2-*Mstnb* 3′ UTR mut and miR-181b-5p. Bar: mean ± SEM. ^***^P < 0.01, One-way ANOVA analysis, *n* = 3–5.

To further verify the miR-181 expression pattern, we attempted to clone the miR-181 family, but the results showed that only miR-181b-5p was cloned in tilapia (Figure 3A and Data Sheet S1). Therefore, miR-181b-5p was chosen for further study. Subsequently, the results of tissue distribution showed that miR-181b-5p had very high expression in the diencephalon and a lower expression in the telencephalon, spinal cord, adipose tissue, white muscle, and kidney (Figure 3B).

As PITA predicted, the seed sequence of miR-181b-5p is completely complementary to the *Mstnb* 3′ UTR at 307–313 nt and 1022–1029 nt (Figure 3C). To determine whether miR-181b-5p directly binds to the 3′ UTR of *Mstnb*, nucleotides TGT were converted to ACA at both sites by site-directed mutagenesis (Figure 3C). Dual luciferase assay results showed that the wild-type *Mstnb* 3′ UTR dramatically downregulated the Rluc/Fluc, while mutagenesis of the *Mstnb* 3′ UTR could reverse miR-181b-5p-induced suppression (Figure 3D).

### The Development of a Polyclonal Antibody Against rMSTNb

**Figure 4:**
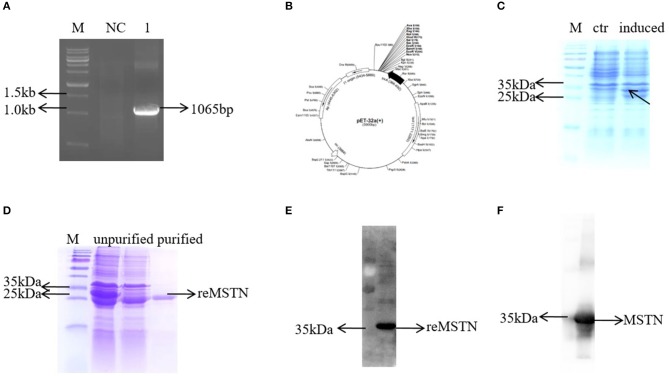
Development of the MSTNb anti-rabbit antibody. **(A)** The *mstnb* ORF was cloned by PCR. **(B)** Schematic diagram of recombinant plasmid pET32a-MSTNb. **(C)** The induced expression of recombinant protein. **(D)** Purification of recombinant protein. **(E)** The purified protein was verified by western blot. **(F)** Mstnb anti-serum was detected, dilution ratio was 1:10,000.

To detect the MSTNb protein level in the following experiment, an antibody against MSTNb was produced (Figure 4). First, the *mstnb* ORF was amplified by PCR and the sequence was confirmed by sequencing (Figure 4A and Data Sheet S1). Then, the amplified fragment was inserted between XhoI and BamHI sites in pET-32a to produce a C-terminal His-tagged rMSTNb protein (Figure 4B). SDS-PAGE revealed that the rMSTNb band with a molecular mass of 35 kDa was expressed successfully in IPTG-induced pET-32a-rMSTNb-transformed bacteria but not in the pET-32a-transformed control bacteria (Figure 4C and Data Sheet S1). After that, the rMSTNb was purified by incising the target strip in the albumen gel, and the results of SDS-PAGE and western blot showed that a 35 kDa protein band can be detected (Figures 4D,E and Data Sheet S1), which suggested that the rMSTNb protein was purified. The purified rMSTNb was used to induce production of polyclonal antibodies in serum of rabbit. To confirm the affinity, western blot was performed and showed that rMSTNb could be detected at the dilution of 1:10,000 (Figure 4F and Data Sheet S1).

### The Isolation and Identification of Primary Muscle Cells From Tilapia

**Figure 5:**
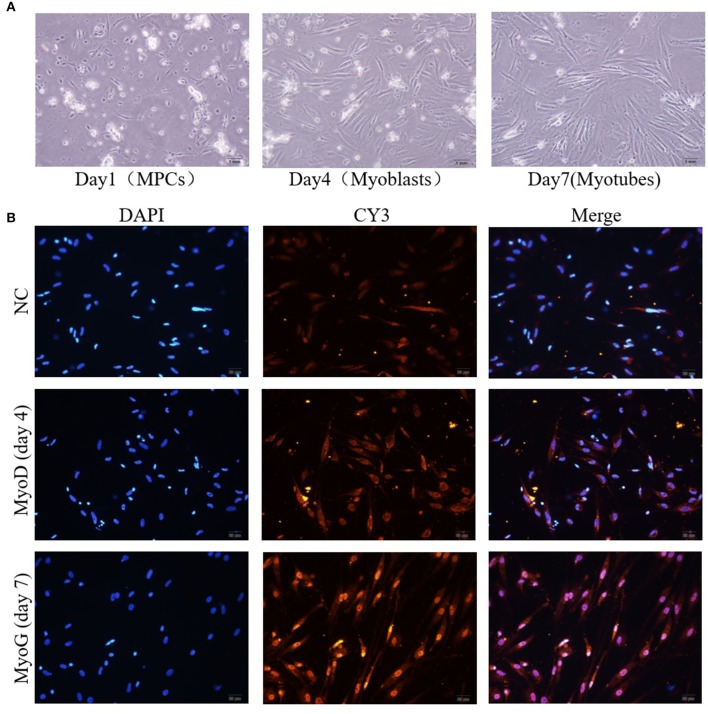
Isolation and identification of primary muscle cells from tilapia. **(A)** The culture of myosatellite cells (day 1), myoblasts (day 4), and myotubes (day 7). **(B)** Immunocytochemical staining of myoblasts (day 4) and myotubes (day 7).

To verify whether miR-181b-5p regulates the expression of Mstnb *in vitro*, a piece of technology for separating the tilapia primary muscle cells was established (Figure 5A). The myosatellite cells were separated and were round on day 1; they then differentiated into spindle-shaped cells called myoblasts on day 4, and they ultimately turned into myotubes on day 7 (Figure 5A). Additionally, the myosatellite cells were verified using immunofluorescence (Figure 5B). Myoblasts expressed MyoD1 during the proliferation phase (day 4), and differentiated myotubes expressed Myogenin at day 7 (Figure 5B).

### miR-181b-5p Silencing Activates *Mstnb* and Inhibits Downstream Genes

**Figure 6:**
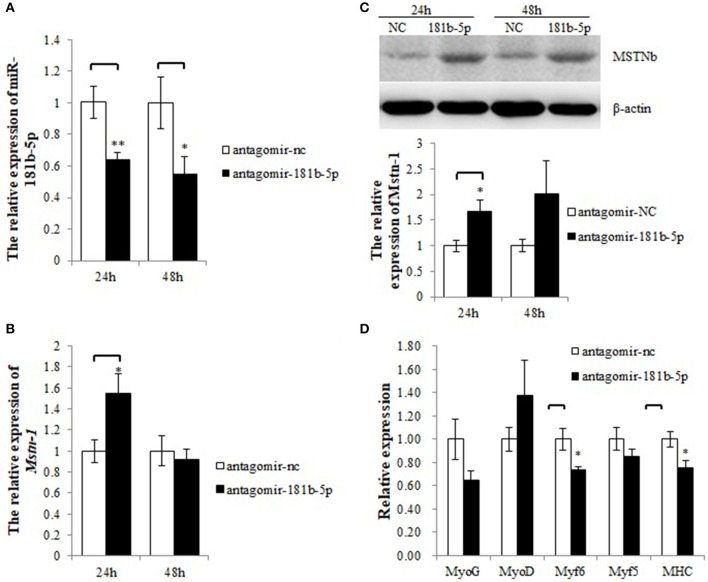
miR-181b-5p silencing influences the expression of Mstnb and its downstream genes. **(A)** The relative expression of miR-181b-5p. **(B)** The mRNA expression of Mstnb. **(C)** The protein level of Mstnb. **(D)** The mRNA expression of Mstnb downstream genes. Bar: mean ± SEM. ^*^P < 0.05 and ^**^P < 0.01, One-way ANOVA analysis, *n* = 3–4.

As *Mstnb* was directly targeted by miR-181b-5p, we hypothesized that the upregulation of MSTNb by antagomir-181b-5p would inhibit the expression of MRFs. In tilapia primary muscle cells, knockdown of miR-181b-5p resulted in a decrease in miR-181b-5p expression at 24 h and 48 h (Figure 6A), while *Mstnb* mRNA was upregulated at 24 h but not at 48 h after the administration (Figure 6B). Knockdown of miR-181b-5p upregulated MSTNb protein levels with a significant difference at 24 h, but no significant difference was noted at 48 h (Figure 6C, Data Sheet S1, Figures S2–S4 and Presentation S1). Thus, 24 h was determined as the administration time. Meanwhile, the mRNA expression of *MHC* and *Myf6* was downregulated by antagomir-181b-5p compared to antagomir-nc after 24 h. However, *myf5, myoG*, and *myoD* mRNA exhibited no change after the knockdown of miR-181b-5p (Figure 6D).

### miR-181b-5p Overexpression Inhibits *Mstnb* and Activates Downstream Genes

**Figure 7:**
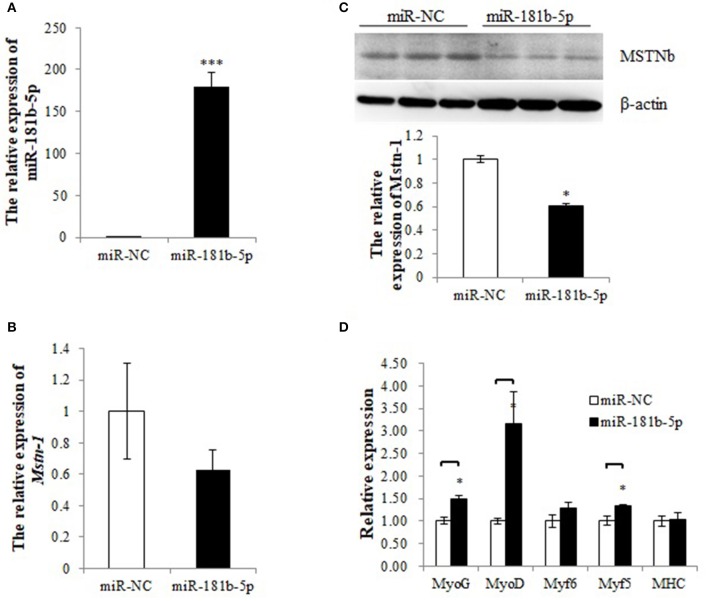
miR-181b-5p overexpression influences the expression of Mstnb and its downstream genes. **(A)** The relative expression of miR-181b-5p. **(B)** The mRNA expression of Mstnb. **(C)** The protein level of Mstnb. **(D)** The mRNA expression of Mstnb downstream genes. Bar: mean ± SEM. ^*^P < 0.05 and ^***^P < 0.01, One-way ANOVA analysis, *n* = 3–4.

To determine whether the miRNA mimics could be transfected into tilapia primary muscle cells, CY3-labeled miR-181b-5p was synthesized and the transfection efficiency was determined by a fluorescence microscope (Figure S1). A total of 59% of the dispersed muscle cells were transfected with the CY3-labeled miR-181b-5p, while the transfection efficiency of the control was 0%. The relative expression of miR-181b-5p was increased thousands of times after transfection (Figure 7A). Overexpression of miR-181b-5p did not affected the mRNA expression level of *mstnb* (Figure 7B), but it decreased MSTNb protein expression (Figure 7C, Data Sheet S1, Figures S5–S7 and Presentation S1). As expected, the mRNA expression of *myf5, myoD*, and *myoG* was upregulated by miR-181b-5p mimics, while *myf6* and *MHC* mRNA expression was not affected (Figure 7D). This contrasted with the results of miR-181b-5p inhibition.

## Discussion

Myostatin has been regarded as a muscle negative regulator in mammals since its discovery (2); its function in muscle development (2), adipogenesis (30), and insulin sensitivity (31) was revealed by several studies. Due to its importance in growth and metabolism, the precise regulation of *Mstn* protein levels is necessary. The regulation of *Mstn* at the transcriptional, post-transcriptional, and translational levels was documented widely. MiRNAs could directly degrade or inhibit the translation of targeted mRNAs at the post-transcriptional level, and they were found to take part in the development of skeletal muscle. In this study, we found that miR-181b-5p may regulate muscle growth of tilapia by targeting *Mstnb*.

In our study, the 3′ UTRs of *mstna* and *mstnb* were cloned, but only *mstnb* was chosen for further study as *mstna* mRNA was only expressed in the brain of tilapia whereas *mstnb* mRNA was expressed in the brain and skeletal muscle. The tissue distribution pattern of tilapia *mstnb* was similar to that of previous studies; RT-PCR results showed that *mstn* was expressed in the muscle, eye, gill, gonad, gut, and brain of Nile tilapia (4). In rainbow trout, both transcripts (*mstn 1a* and *mstn 1b*) were present in the muscle, testes, eye, brain, and spleen (32). In *Trachidermus fasciatus*, the *mstn* was highly expressed in the muscle and intestine and weakly expressed in the brain and liver (33). Although *mstn* is widely distributed in various tissues of teleosts, it is highly expressed in muscle (4, 34). We hypothesized that *mstnb* was important for the growth of skeletal muscle in tilapia.

The relative luciferase activity of *mstnb* was downregulated by several miRNAs, including miR-30a-3p, miR-181a/b-5p, miR-27a/b, miR-206-3p, miR-200a, and miR-455b. This is consistent with the fact that one gene may be targeted by multiple miRNAs and one miRNA may target several genes (35). In addition, the other members of the miR-181 family were also shown to target the *mstnb* 3′ UTR since their seed sequences are identical. These results suggested that miRNAs targeted mRNA by interacting with the seed sequences (36, 37). Although only miR-181b-5p was investigated in this study, other miRNAs might also play a role in regulating *mstn* at the post-transcriptional level. For example, miR-27a was reported to target *mstn* and induce the differentiation of C2C12 (20), and miR-27a/b regulates MSTN expression through negative feedback auto-regulation in mice (21). MiR-206, a miRNA that is abundantly expressed in skeletal muscle that modulates the development and disease of skeletal muscle in mammals (38), was also shown to decrease the luciferase activity of *mstnb* in tilapia. In addition, miR-206 was shown to target the 3′ UTR of *mstn* in Texel sheep, a sheep known for its double muscling (17). The above results suggested that *mstnb* could be regulated by several miRNAs in both mammals and teletosts.

Among those miRNAs, miR-181 was reported to associate with the TGF-β superfamily and regulate Hox-A11 expression in mammals (29). Meanwhile, only miR-181b-5p, a member of the miR-181 family from tilapia, was cloned. We focused on miR-181b-5p in the subsequent research. The results of tissue distribution showed that miR-181b-5p was expressed predominantly in the diencephalon, and smaller amounts in the telencephalon, liver, adipose, white muscle, and kidney, which was in line with the studies that showed that miR-181 was widely expressed across tissues and played a vital role in the immune system (39), skeletal muscle growth (29), hemopoiesis (40), brain ischemia (41), and so on. In addition, the expression profiles of miR-181 were correlated with the development stage (42) and nutrition status (43). Therefore, the development stage may be the cause of low abundance of miR-181b-5p expression in white muscle.

Furthermore, the results of our study showed that miR-181b-5p could target *mstnb* using a mutated reporter in the predicted target sites and a dual-luciferase assays. To further study the possible roles of miR-181b-5p in the post-transcriptional regulation of *mstnb* in tilapia, the primary muscle cells of tilapia were used as cell models to perform the experiments of miR-181b-5p knockdown and overexpression. In our study, double enzyme hydrolysis (collagenase IV and trypsin) was used to digest the muscle of tilapia. The research in fish cell culture developed quickly following Wolf establishing the RTG-2 cell line in rainbow trout (44). However, the culture of primary muscle cells was developed slowly because the myogenic precursor cells only proliferate under certain conditions, such as wound healing, exercise, and disease (45). A method to isolate and culture primary myogenic precursor cells was established in several fish species, including rainbow trout, salmon, and sea bream (46–48). We also cultured primary muscle cells in tilapia using the protocol described by Froehlich et al. (27). This study was the first paper to describe the technology of culturing primary muscle cells from tilapia.

In this study, knockdown of miR-181b-5p in primary muscle cells led to an upregulation of MSTNb protein, and overexpression caused a downregulation effect, indicating that miR-181b-5p might participate in the regulation of MSTNb expression levels *in vitro*. Similar results were obtained using deep sequencing and a dual-luciferase experiment in *S. chuatsi* (23). Meanwhile, the opposite expression pattern between miR-181a-5p and *mstn* in white and red muscle was observed (23). In our study, miR-181b-5p targeting *mstnb* was further confirmed in tilapia primary muscle cells. In addition, miR-181 was reported to regulate the differentiation of myoblasts in mice by targeting Hox-A11, an inhibitor of MyoD (29). Although miR-181 may target different genes in mammals and fish, the function of miR-181 in regulating muscle growth may be conserved in evolution.

Although MSTN was reported to inhibit the growth of skeletal muscle in mammals and some fishes, the regulatory role of MSTN on the growth of skeletal muscles in tilapia was not reported. In the present study, the mRNA expression of MRFs was detected after stimulating or inhibiting the expression of MSTNb by antagomir-181b-5p and miR-181b-5p. MRFs play an important role during the proliferation and differentiation of skeletal muscle cells. Lacking both *MyoD* and *Myf5* in skeletal muscle stem cells caused the accumulation of satellite cell progeny in damaged muscle and blocked differentiation (49). *MyoD* and *MyoG* can induce the transcription of Myomaker and promote the fusion of myoblasts, which is an important step during skeletal muscle differentiation (50). It was also reported that miR-374b directly targeted *Myf6* and inhibited the differentiation in C2C12 (51). It suggested that the expression of MRFs may reflect the differentiation of muscle cells. Except for MRFs, MSTN knockdown by small hairpin RNA (shRNAs) led to sustained cell proliferation of myoblasts and upregulated expression of *Myf6* in goats (52). Our study showed that the mRNA expression of MRFs was decreased when MSTNb was promoted by antagomir-181b-5p and increased when MSTNb was inhibited by miR-181b-5p. These results suggested that MSTNb may regulate muscle growth by modulating the expression of MRFs in tilapia, which was consistent with those reported in mammals (53–56).

Since MSTN is a negative regulator, inhibition or mutation of this gene may be useful for muscle development (55). For instance, knocking down *mstn* using siRNA increased muscle mass in mice (57), and knocking out *mstnb* using TALEN (transcription activator-like effector nucleases) also induced muscle hyperplasia and body weight increase in zebrafish (58). In their study, the circumferences and body weights of *mstnb*-deficient zebrafish increased after 80 days post-fertilization (58). Similarly, primary muscle cells treated with human recombinant MSTN (huMSTN) resulted in a myotube diameter decreased of up to 20% (59). The mRNA expression of *Myf5, MyoD*, and *MyoG* was significantly increased after rainbow trout primary myosatellite cells were treated with MSTN-1 (7), which was inconsistent with our study. It is noteworthy that the treatments were different. In their study, primary myosatellite cells were incubated with MSTN-1 after culturing 72 h for 3 days or 7 days (7), while in our study, MSTNb was indirectly increased by antagomir after culturing over 1 day (for 24 h). Obviously, the mechanism of MSTN-1 and antagomir treatment of myosatellite cells is different. In addition, the expression of MRFs is dynamic during the differentiation of skeletal muscle. The expression of Myf5 and MyoD was increased in proliferating myoblasts, while MyoG and Myf6 increased in the terminally differentiated myotubes (28). It is hard to compare the two studies because the experimental conditions were different. Despite this, we hypothesized that *mstnb* participates in the regulation of muscle growth in tilapia in a similar way in fishes as in mammals.

In this study, miR-181b-5p overexpression inhibited MSTNb and activated downstream gene expression. Our results suggest that miR-181b-5p may regulate the muscle growth of tilapia by targeting *myostatin b* in tilapia.

## Data Availability Statement

The datasets generated for this study are available on request to the corresponding author.

## Ethics Statement

The Tilapia used in this study were obtained from the local farm of Guangdong Tilapia Breeding Farm, Guangzhou, China. No specific permissions are required for the buying of fish. All animal experiments were performed with the approval of the Sun Yat-Sen University Animal Care and Use Committee and in full compliance with its ethical guidelines.

## Author Contributions

WL and ZZ conceived and designed the experiments. ZZ, XY, JJ, and GY performed the experiments. ZZ and WL analyzed the data. ZZ and CS wrote the paper. All authors read and approved the final manuscript.

### Conflict of Interest

The authors declare that the research was conducted in the absence of any commercial or financial relationships that could be construed as a potential conflict of interest.

## Supplementary Material

Figure S1 Transfection efficiency of tilapia muscle pituitary cells by CY3-labeled miR-181b-5p. DAPI, cells were dyed by DAPI and image taken with 358 nm exciting laser; CY3, image taken with 550 nm exciting laser; MERGE, image merged by the two images DAPI and CY3.

Figure S2 Western bolt original image of β-actin corresponding to Figure 6C.

Figure S3 Western bolt original image of Mstnb corresponding to Figure 6C.

Figure S4 Coomassie-blue staining of SDS-PAGE corresponding to Figure 6C.

Figure S5 Western bolt original image of Mstnb corresponding to Figure 7C.

Figure S6 Western bolt original image of β-actin corresponding to Figure 7C.

Figure S7 Coomassie-blue staining of SDS-PAGE corresponding to Figure 7C.

Table S1 Primers used in this research.

Data Sheet S1 Agarose gel original images corresponding to Figures 1A,B, 3A, 4A. Coomassie-blue original images corresponding to Figures 4C,D. Western bolt original images corresponding to Figures 4E,F, 6C, 7C.

Presentation S1 Coomassie-blue staining and Western bolt original images corresponding to Figures 6C, 7C.
