# Supplementary figures and images for: miR-181b-5p May Regulate Muscle Growth in Tilapia by Targeting Myostatin b
Source: Front Endocrinol (Lausanne). 2019 Dec 3;10:812. doi: 10.3389/fendo.2019.00812 (PMC6902659; doi:10.3389/fendo.2019.00812)

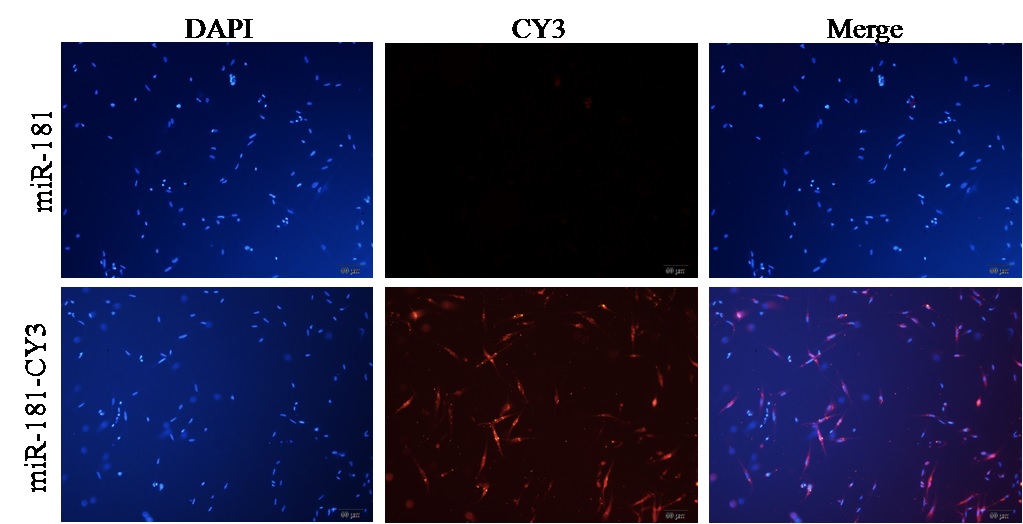

Supplement: Figure S1 — Transfection efficiency of tilapia muscle pituitary cells by CY3-labeled miR-181b-5p. DAPI, cells were dyed by DAPI and image taken with 358 nm exciting laser; CY3, image taken with 550 nm exciting laser; MERGE, image merged by the two images DAPI and CY3. [file Image_1.JPEG]

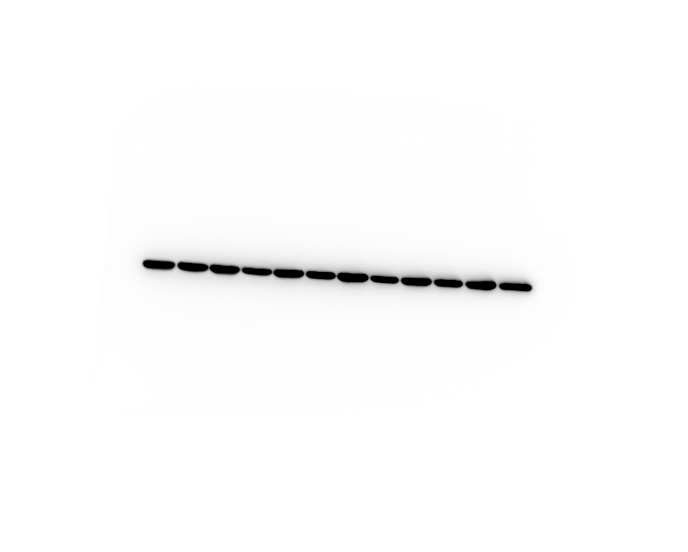

Supplement: Figure S2 — Western bolt original image of β-actin corresponding to Figure 6C. [file Image_2.JPEG]

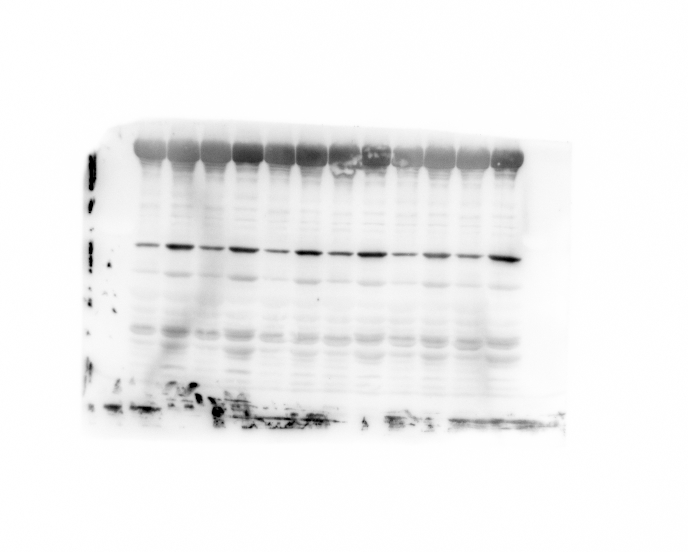

Supplement: Figure S3 — Western bolt original image of Mstnb corresponding to Figure 6C. [file Image_3.JPEG]

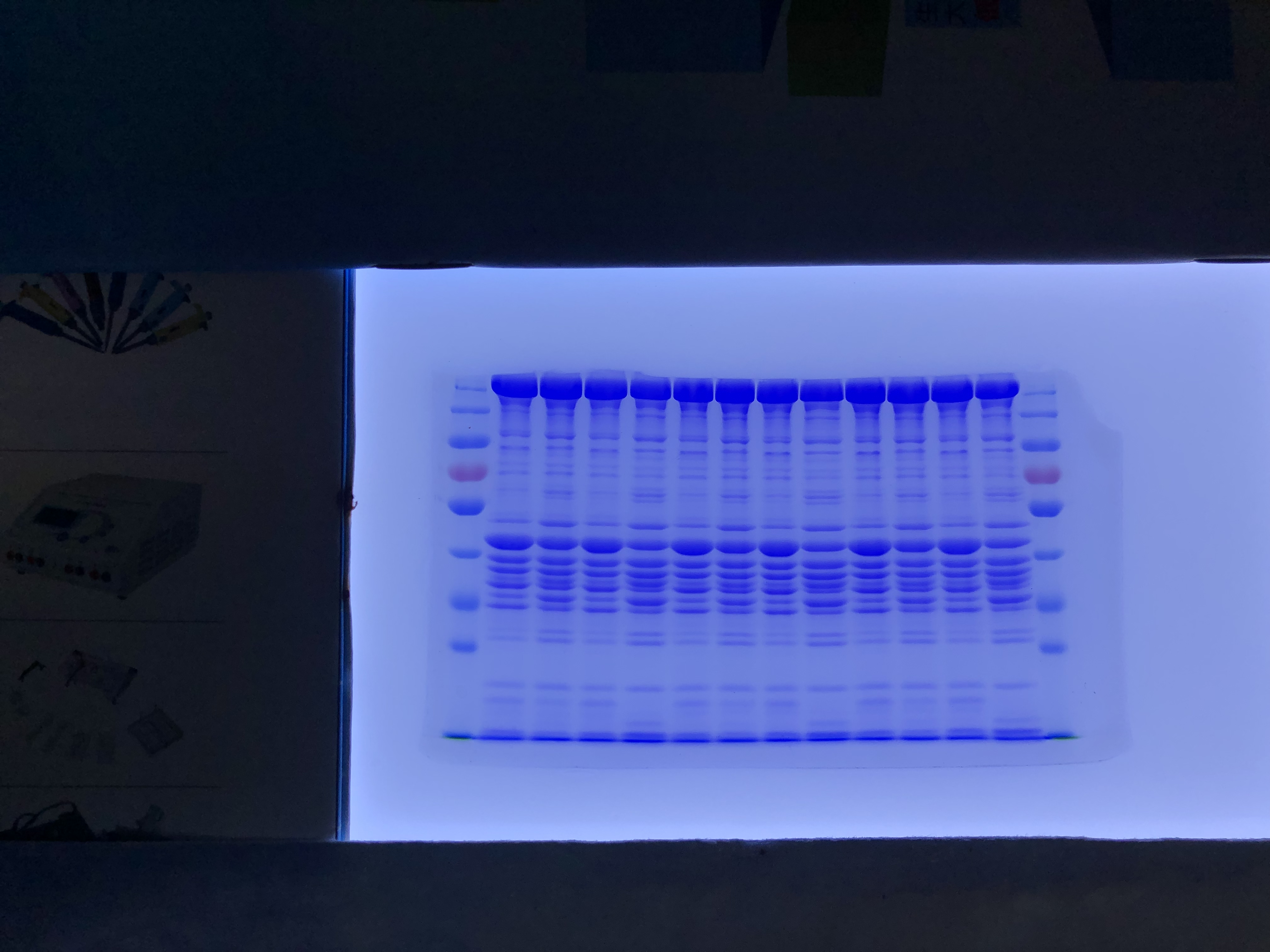

Supplement: Figure S4 — Coomassie-blue staining of SDS-PAGE corresponding to Figure 6C. [file Image_4.JPEG]

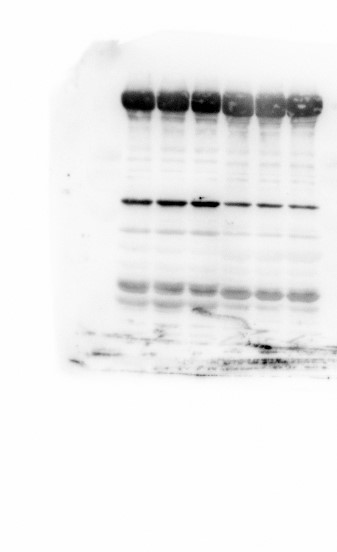

Supplement: Figure S5 — Western bolt original image of Mstnb corresponding to Figure 7C. [file Image_5.JPEG]

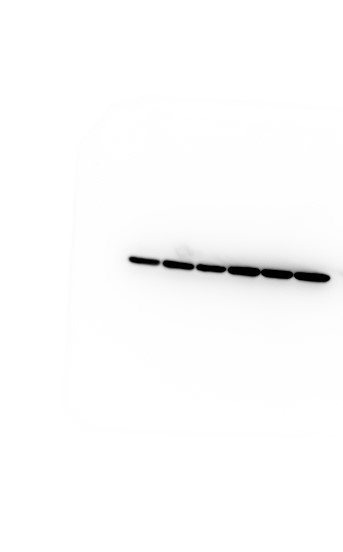

Supplement: Figure S6 — Western bolt original image of β-actin corresponding to Figure 7C. [file Image_6.JPEG]

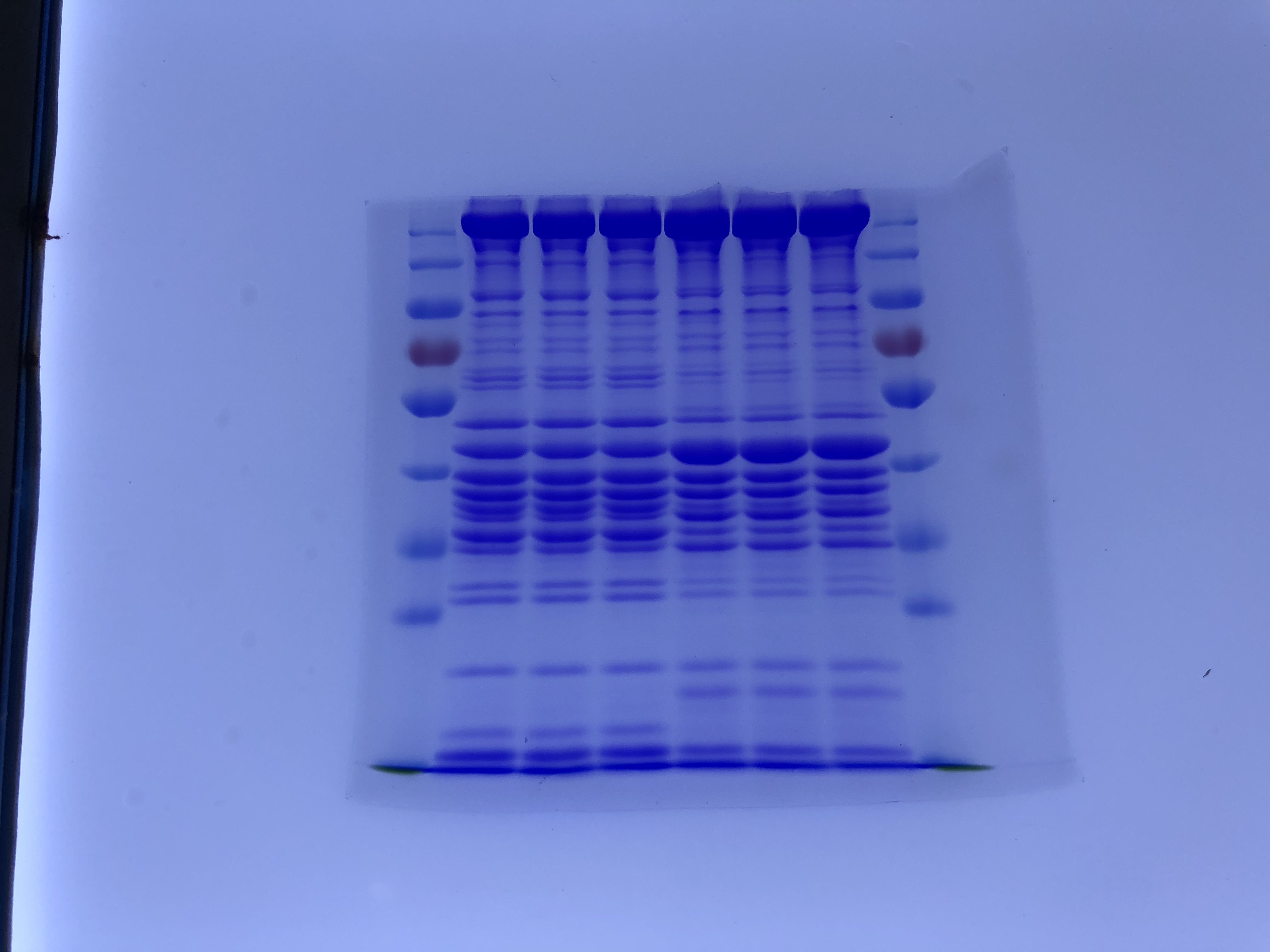

Supplement: Figure S7 — Coomassie-blue staining of SDS-PAGE corresponding to Figure 7C. [file Image_7.JPEG]

Fig 1A


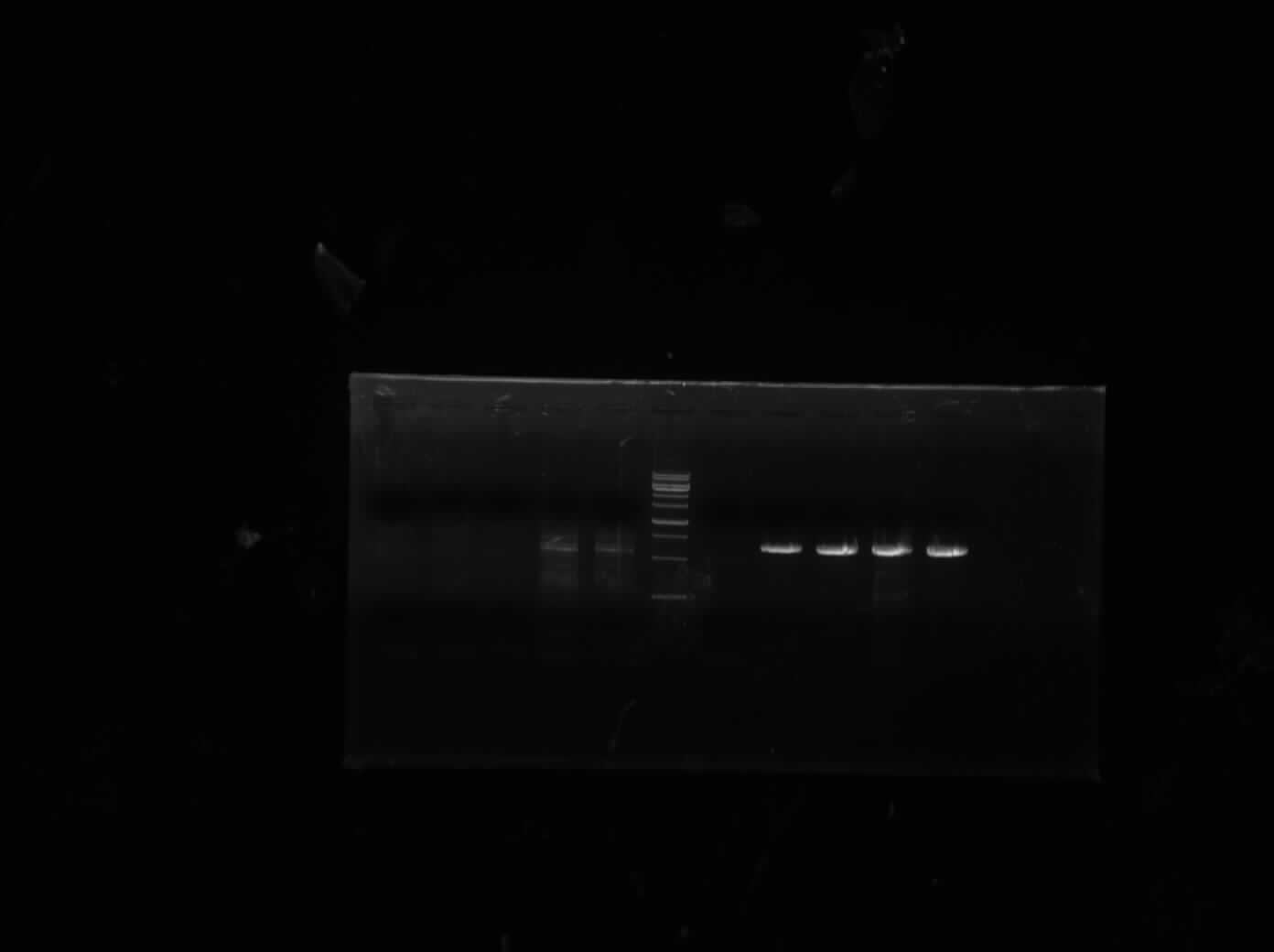


Fig 1B


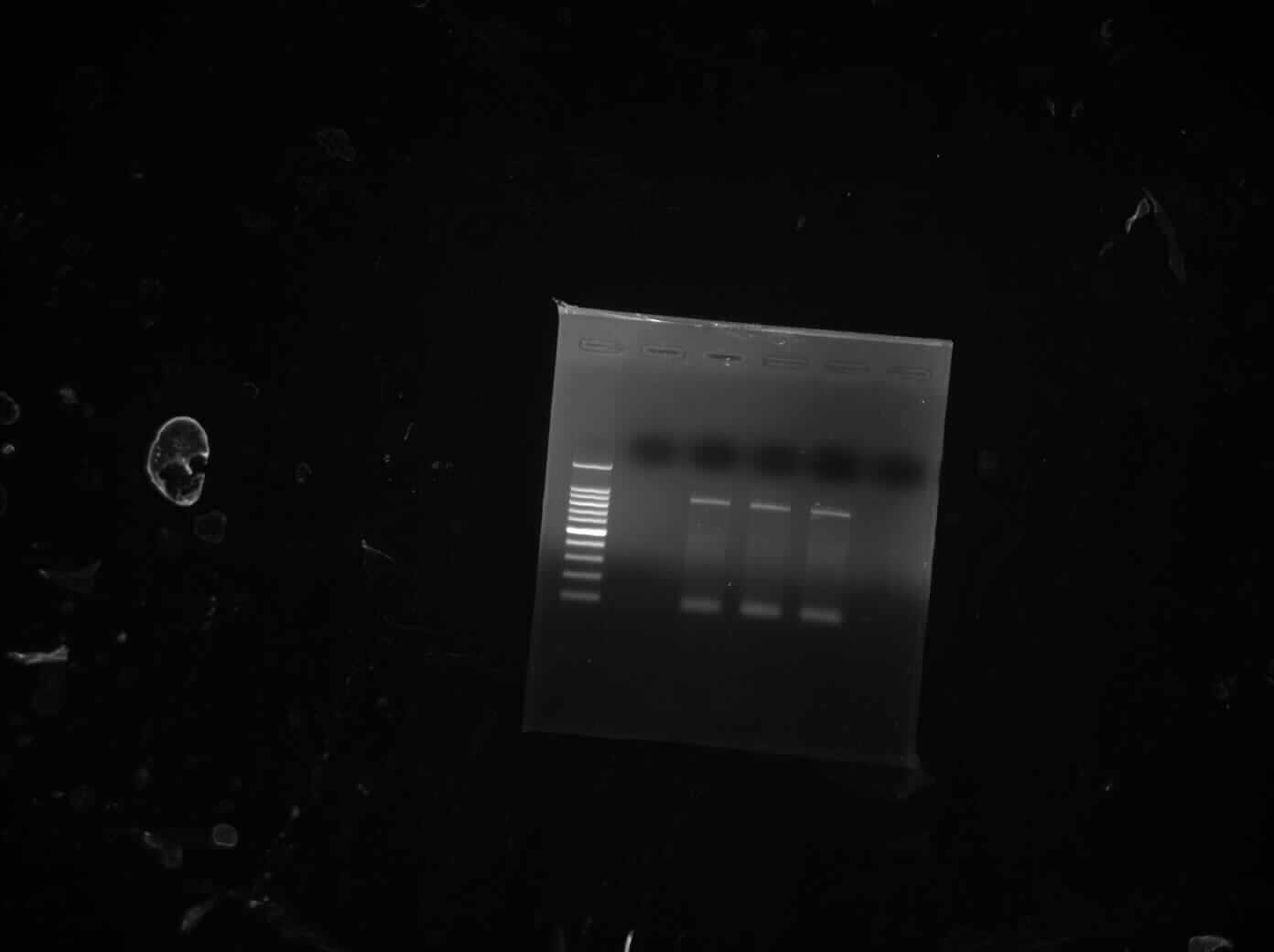


Fig 3A


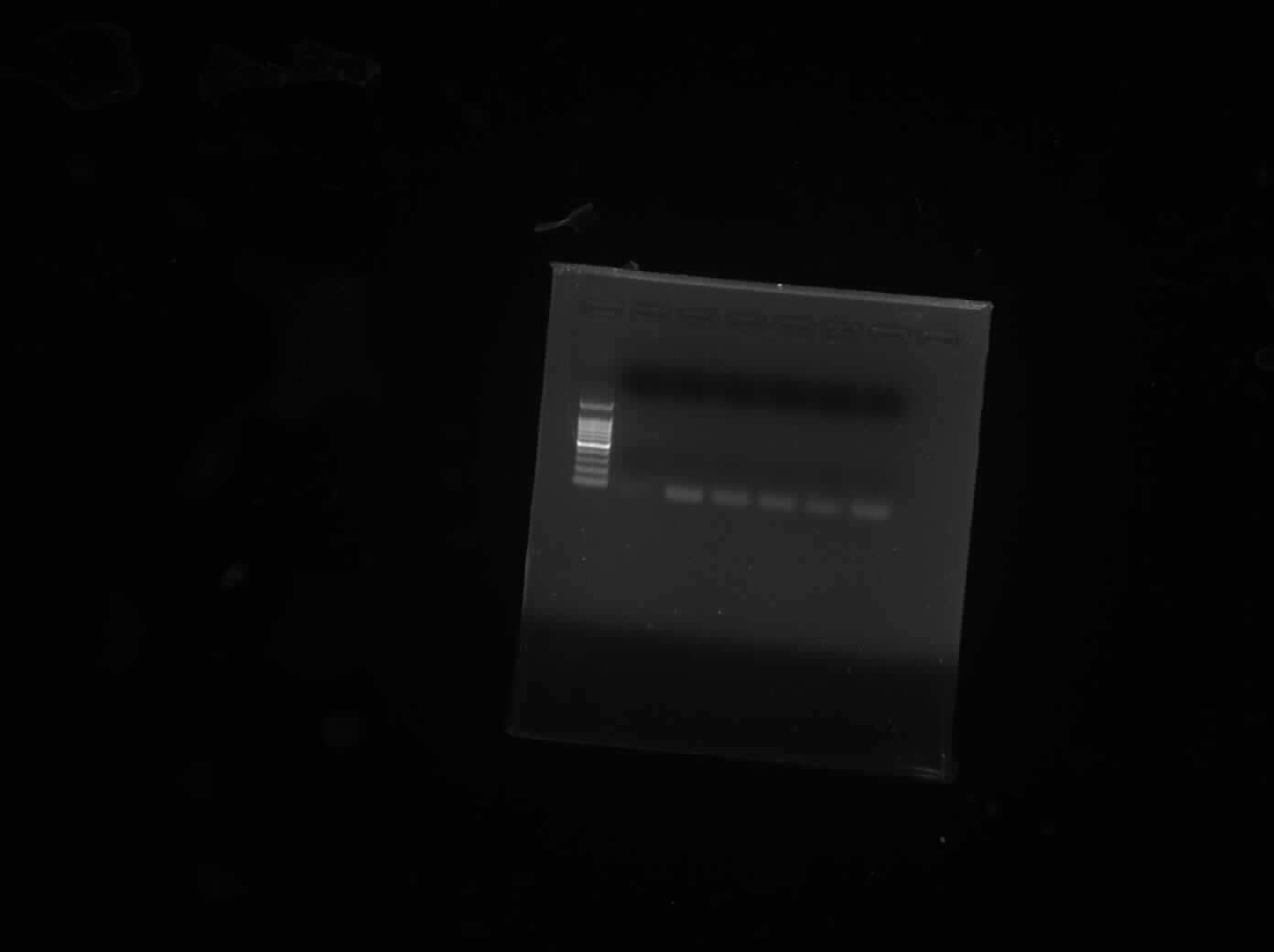


Fig 4A


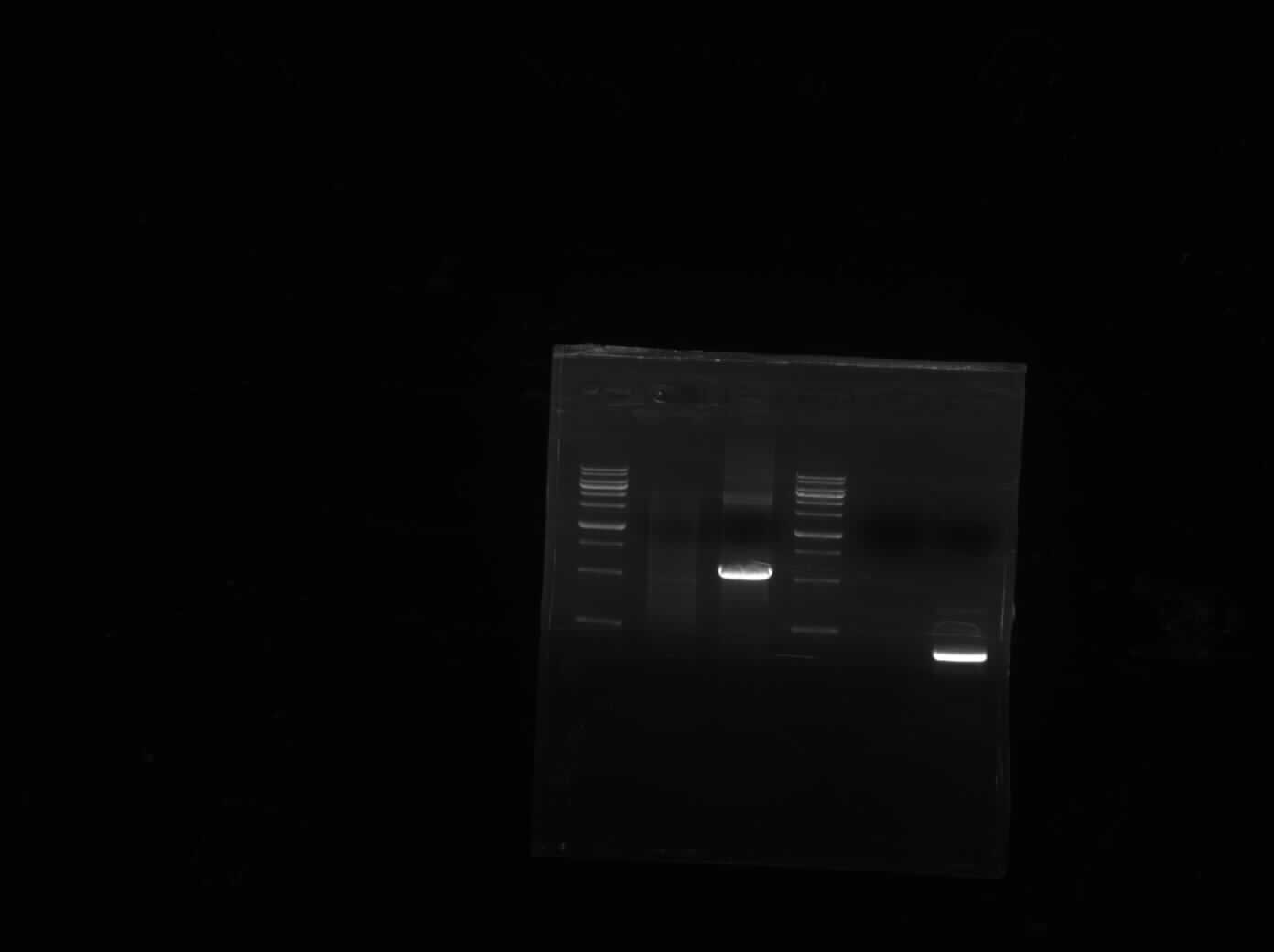
Fig 4C


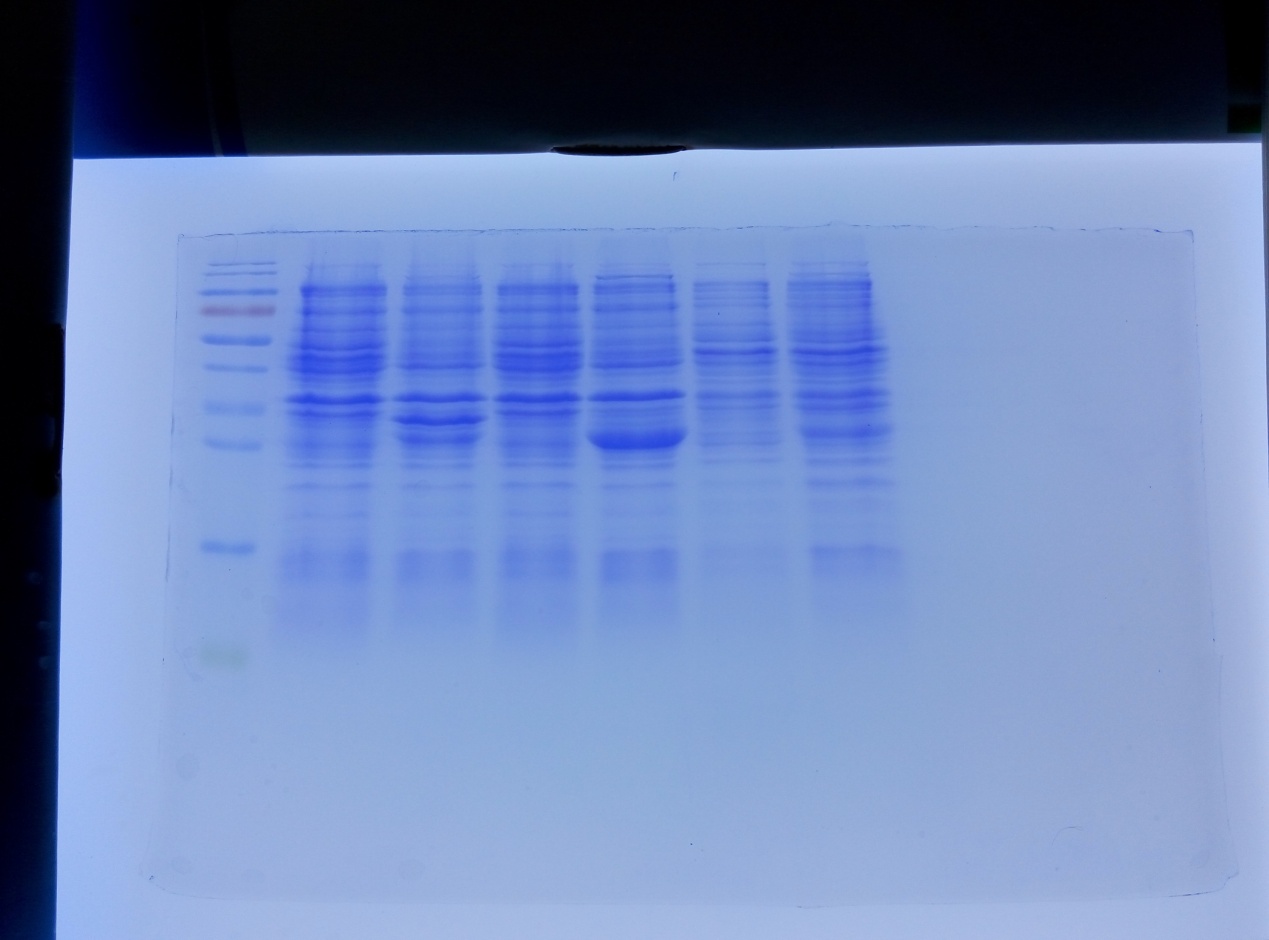


Fig 4D


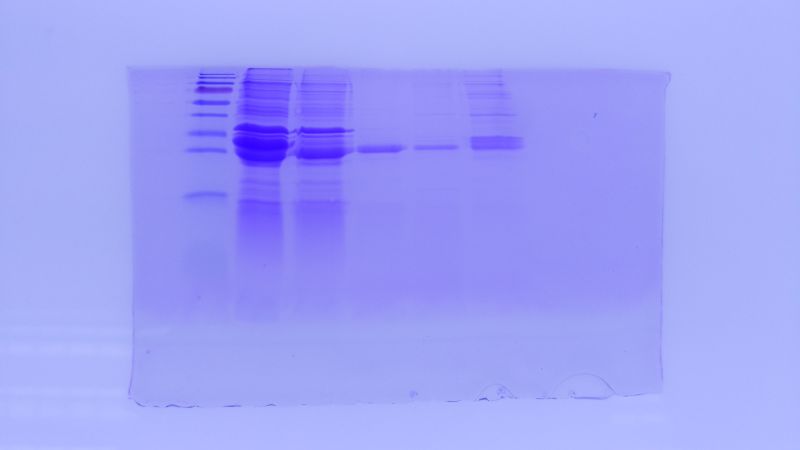


Fig 4E


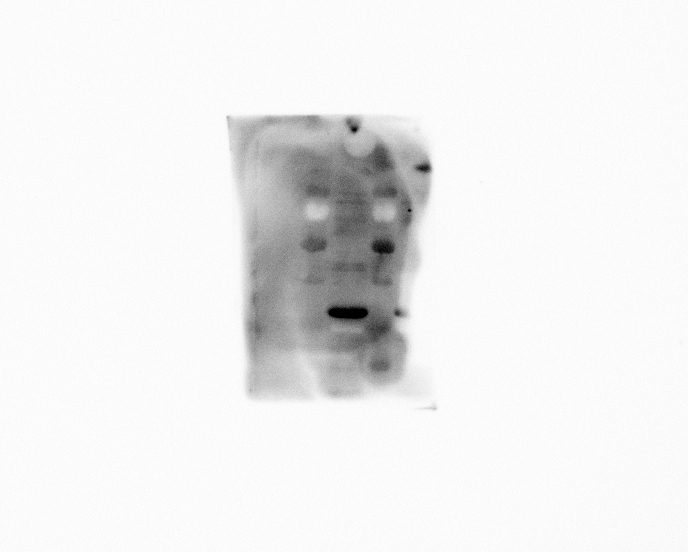


Fig 4F


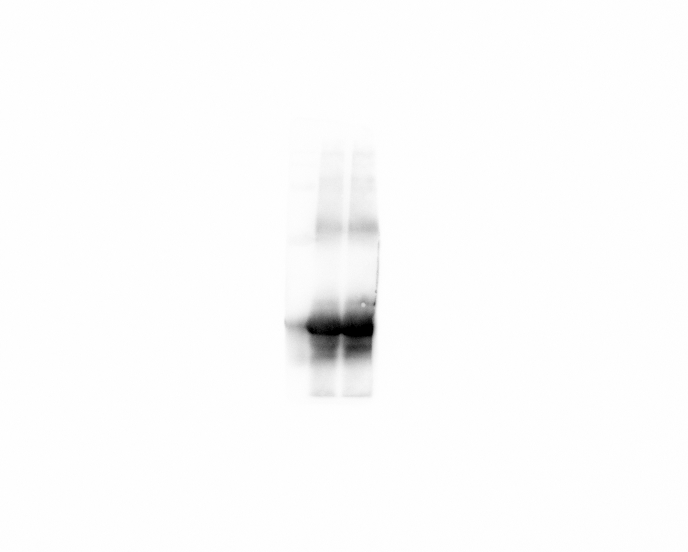


Fig 6C


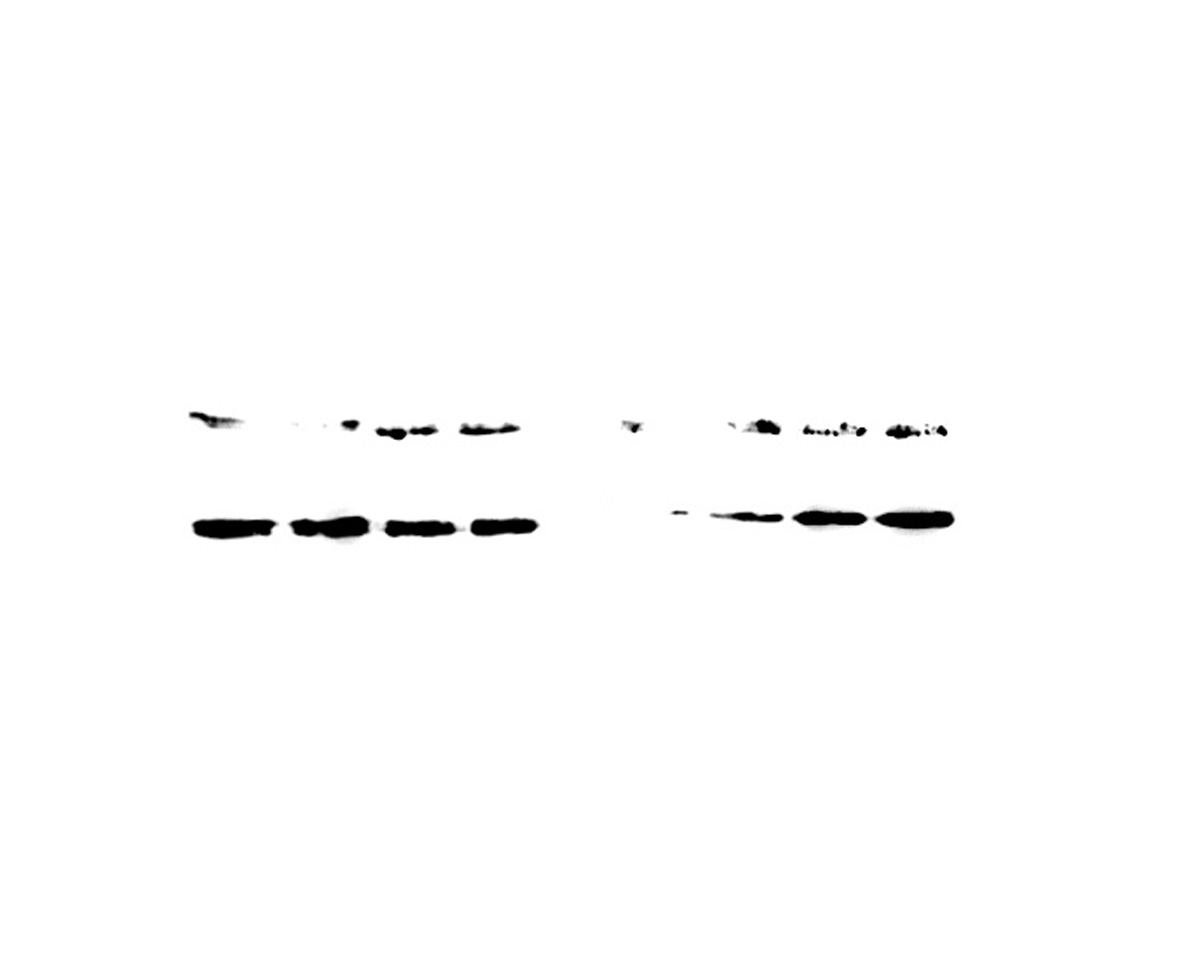


Fig 6C


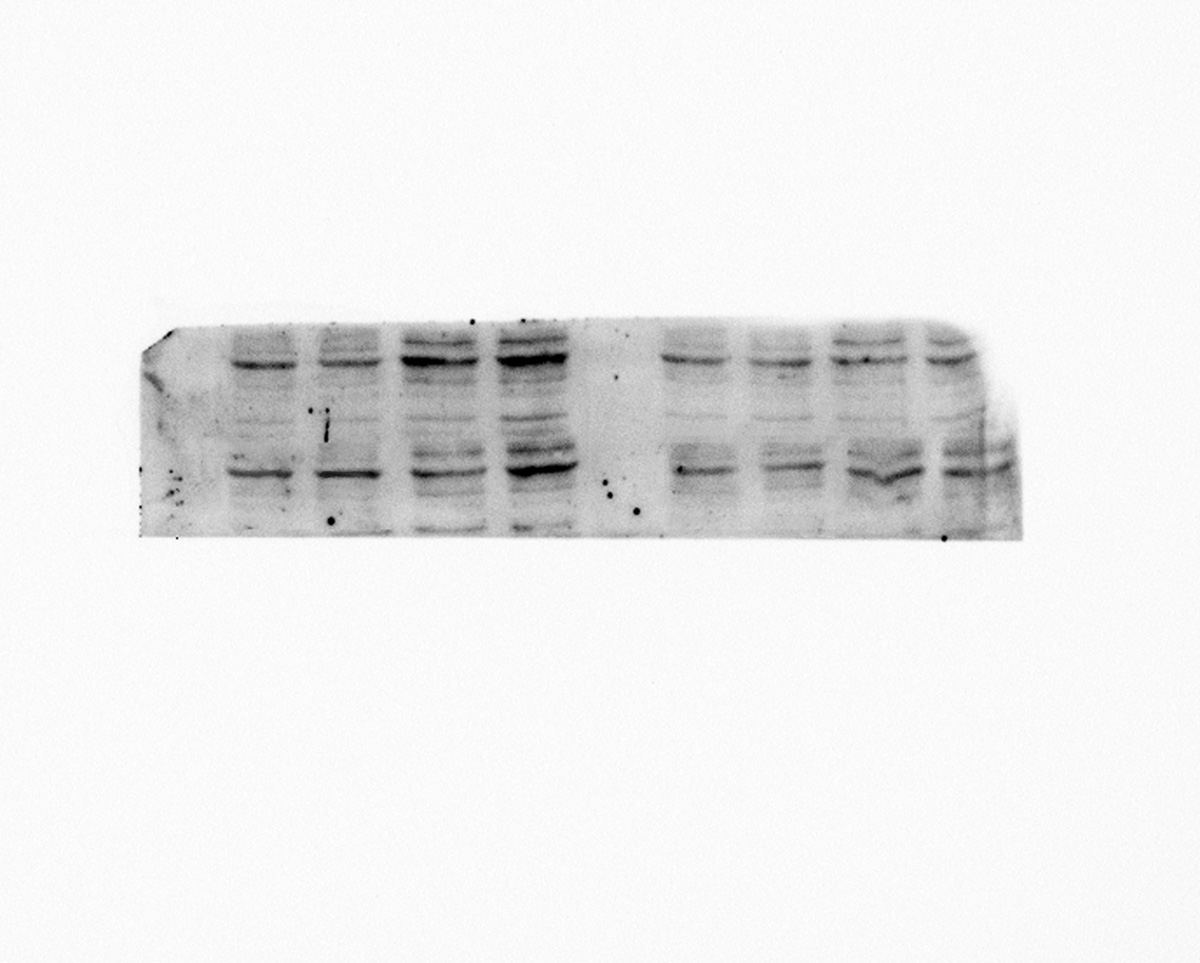


Fig 7C


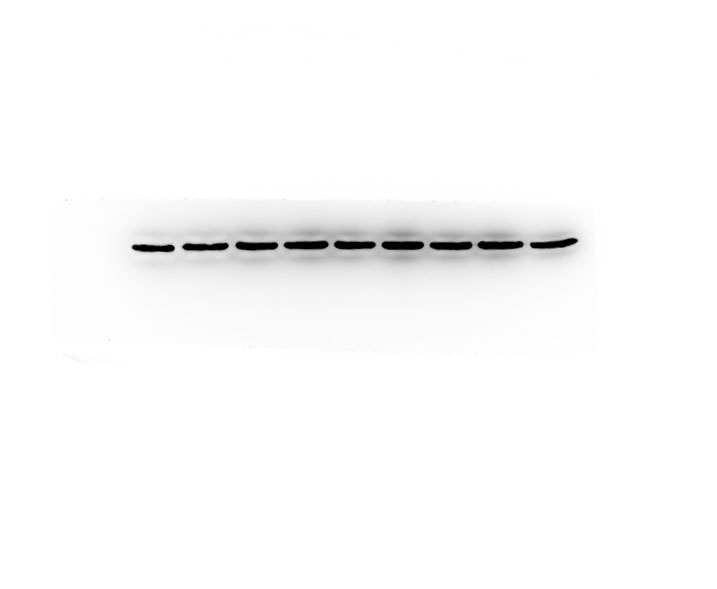


Fig 7C


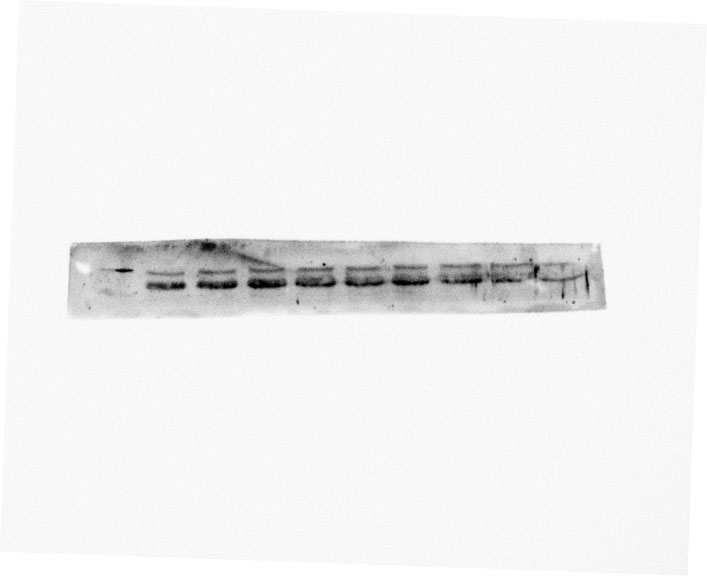

Supplement: Data Sheet S1 — Agarose gel original images corresponding to Figures 1A,B, 3A, 4A. Coomassie-blue original images corresponding to Figures 4C,D. Western bolt original images corresponding to Figures 4E,F, 6C, 7C. [file Data_Sheet_1.docx]
