# Supplementary material for: miR-181b-5p May Regulate Muscle Growth in Tilapia by Targeting Myostatin b
Source: Front Endocrinol (Lausanne). 2019 Dec 3;10:812. doi: 10.3389/fendo.2019.00812 (PMC6902659; doi:10.3389/fendo.2019.00812)
Supplement: Table S1 — Primers used in this research. [file Table_1.DOC]

Table. s1. Primers used in this research

| primers | sequences(5'-3') |  |
| --- | --- | --- |
| primers for reverse transcription | | |
| miR-181b-5p-RT | CTCAACTGGTGTCGTGGAGTCGGCAATTCAGTTGAGACCCACC |  |
| U6-RT | GTCGTATCCAGTGCAGGGTCCGAGGTATTCGCACTGGATACGACAAAAAT |  |
| primers for cloning mstn | | |
| UTR-F | TATGGCAAGATCCCCTCCAT |  |
| UTR-R | AGAGTTGCTGCATTACAACATTT |  |
| primers for qRT-PCR | | |
| miR-181b-5p-qF | ACACTCCAGCTGGGAACATTCATTGCTGTC |  |
| U6-F | GCGCGATTCGTGAAGCGTTCC |  |
| U6-R | GTGCAGGGTCCGAGGT |  |
| mstn-F | GTCAGCACCCCAGTAGACAC |  |
| mstn-R | CCTTCATTCGCAGCTTGCTC |  |
| Myf5-F | GAGCTGCTACGAGAACAGGT |  |
| Myf5-R | CGGCTGCTTTATCATCCACG |  |
| Myf6-F | CCAGAGGCTACCCAAAGTGG |  |
| Myf6-R | AAGTGGAATGGTCAGCGGAG |  |
| MyoD-F | ACGCCATCAGCTACATCGAG |  |
| MyoD-R | ACAGGTGGGGCCGTTAAAAT |  |
| MyoG-F | GCTGATGAACCCCAACCAGA |  |
| MyoG-R | GGTAGAGCAAACCCTGCTGT |  |
| MHC-F | CCCAACGAGACCAAGACTCC |  |
| MHC-R | GGGCCAATTCTTAACCCCCA |  |
| primers for constructing pET32 vector | | |
| mstn-pF | TGCTCTAGACATCATCATCATCATCATGACCAAGAGGCGCACCAGCA |  |
| mstn-pR | CGCGGATCCTCAGGAGCATCCACAACGGT |  |
| primers for constructing psiCHECK2 vectors | | |
| mstn-wt-F | CCGCTCGAGGTTGGGATGGAGAGAGAAAG |  |
| mstn-wt-R | CGCGGATCCAGAGTTGCTGCATTACAACA |  |
| mstn-mut-F | GCTGGACCTTGAAGTGAAACAAGCCAGAAATGATCACAG |  |
| mstn-mut-R | CTGTGATCATTTCTGGCTTGTTTCACTTCAAGGTCCAGC |  |
| mstn-mut2-F | CAGAAAACGATGTGAAACACGAAATCATGCCTAGAC |  |
| mstn-mut2-R | GTCTAGGCATGATTTCGTGTTTCACATCGTTTTCTG |  |
