# Supplementary material for: miR-181b-5p May Regulate Muscle Growth in Tilapia by Targeting Myostatin b
Source: Front Endocrinol (Lausanne). 2019 Dec 3;10:812. doi: 10.3389/fendo.2019.00812 (PMC6902659; doi:10.3389/fendo.2019.00812)
Supplement: Presentation S1 — Coomassie-blue staining and Western bolt original images corresponding to Figures 6C, 7C. [file Presentation_1.PPTX]

## Slide 1
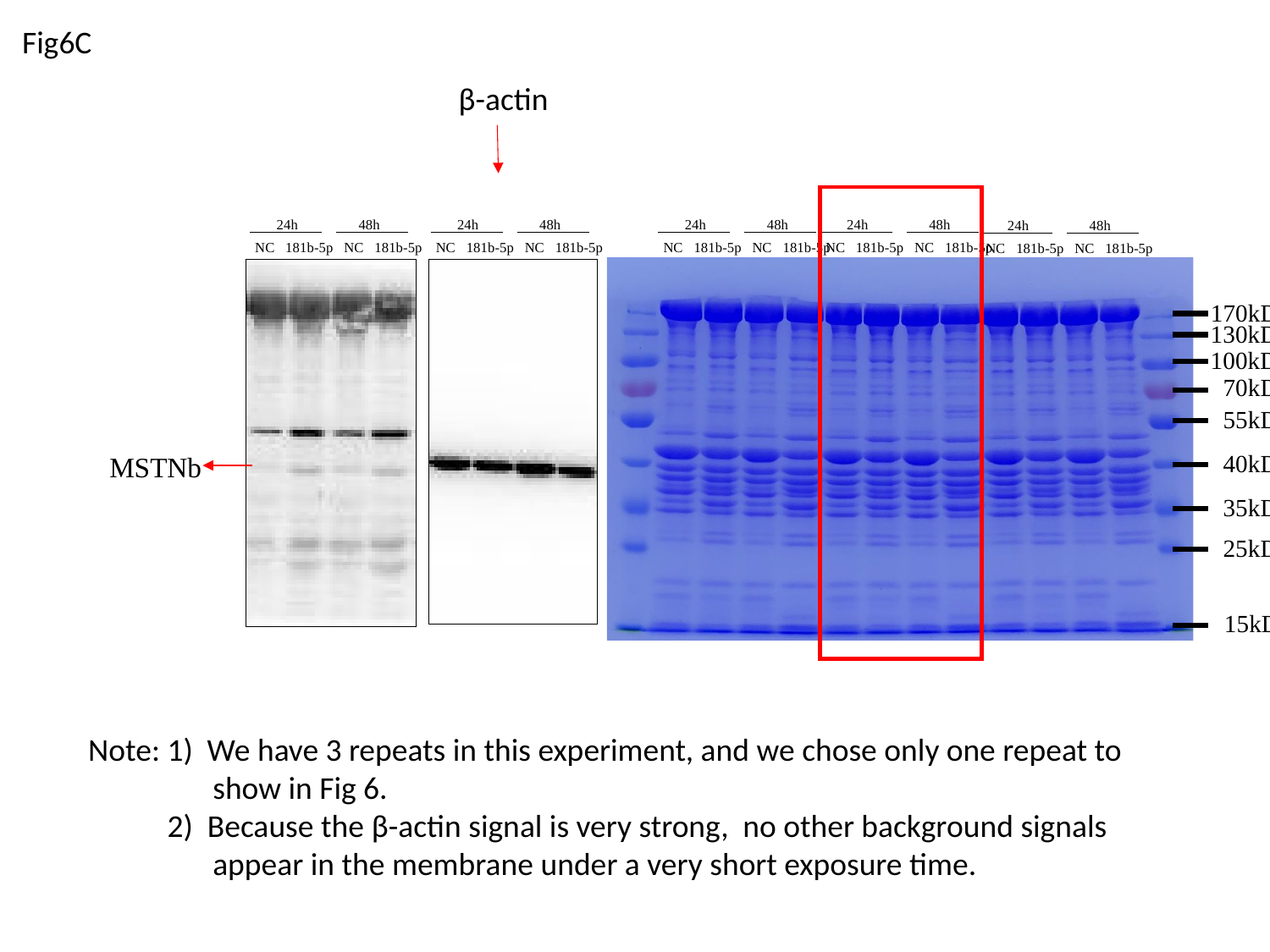

Fig6C
β-actin
 24h 48h
 NC 181b-5p NC 181b-5p
 24h 48h
 NC 181b-5p NC 181b-5p
 24h 48h
 NC 181b-5p NC 181b-5p
 24h 48h
 NC 181b-5p NC 181b-5p
 24h 48h
 NC 181b-5p NC 181b-5p
170kD
130kD
100kD
70kD
55kD
40kD
35kD
25kD
15kD
MSTNb
Note: 1) We have 3 repeats in this experiment, and we chose only one repeat to show in Fig 6.
 2) Because the β-actin signal is very strong, no other background signals appear in the membrane under a very short exposure time.

## Slide 2
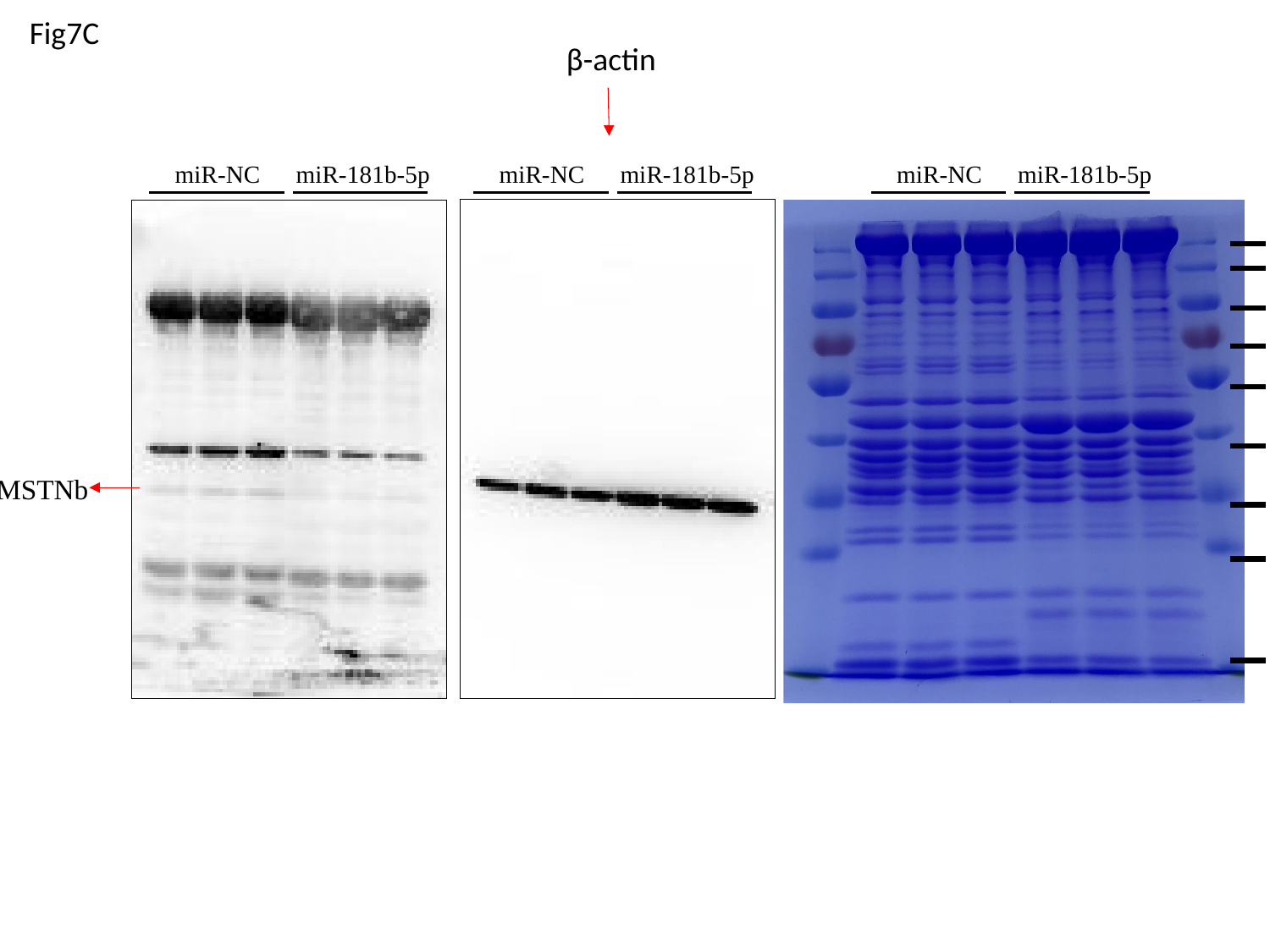

Fig7C
β-actin
miR-NC
miR-181b-5p
miR-NC
miR-181b-5p
miR-NC
miR-181b-5p
170kD
130kD
100kD
70kD
55kD
40kD
35kD
25kD
15kD
MSTNb
